# Comparison of all completed suicides in Frankfurt am Main (Hessen) before and during the early COVID-19 pandemic

**DOI:** 10.1007/s12024-023-00754-8

**Published:** 2023-11-29

**Authors:** S. C. Koelzer, M. A. Verhoff, S. W. Toennes, C. Wunder, M. Kettner, N. Kern, A. Reif, C. Reif-Leonhard, C. Schlang, I. Beig, V. Dichter, N. Hauschild, D. Lemke, S. Kersten, F. Holz

**Affiliations:** 1 Institute of Legal Medicine, University Hospital Frankfurt, Goethe University, Frankfurt am Main, Germany; 2 Institute of Legal Medicine, University Medical Center Mainz, Johannes Gutenberg University, Mainz, Germany; 3 Department of Psychiatry, Psychosomatic Medicine and Psychotherapy, University Hospital Frankfurt, Goethe University, Frankfurt am Main, Germany; 4 Health Department, Frankfurt am Main, Germany; 5 Institute of General Medicine, Goethe University, Frankfurt am Main, Germany; 6 Police Department, Criminal Investigation Department, Fatality Bureau, Frankfurt am Main, Germany

**Keywords:** SARS-CoV-2, Suicide numbers, Vulnerability, Mental disorders, Suicide methods

## Abstract

To research the effect of the COVID-19 pandemic on mental health, the prevalence and characteristics of all completed suicides in the city of Frankfurt am Main were compared for a 10-month period before the pandemic (March 2019–December 2019) with one during the early pandemic (March 2020–December 2020). Medicolegal data collected in the context of the FraPPE suicide prevention project were evaluated using descriptive statistical methods. In total, there were 81 suicides during the early pandemic period, as opposed to 86 in the pre-pandemic period. Though statistically not significant, the proportion of male suicides (73%) was higher during the early pandemic period than before (63%). The age-at-death was comparable in the pre-pandemic and pandemic periods (average, 54.8 vs. 53.1 years). Between these two periods, there was no difference in respect to the three most commonly used suicide methods by men: fall from a height (26% vs. 22%), intoxication, and strangulation (each 24% vs. 19%). For women, there was, however, a shift in methods from strangulation (38%), intoxication (28%), and fall from a height (19%) to fall from a height (50%), strangulation (18%), intoxication, and collision with a rail vehicle (14% each). There was a trend towards more suicides among non-German nationals during the early pandemic (suicide rate/100,000 inhabitants: German, 14.3 vs. 11.5; non-German, 4.4 vs. 8.8). Before the pandemic, 54% of the suicides were known to have a mental illness in contrast to 44% during the early pandemic. Overall, no increase in completed suicides could be observed in Frankfurt am Main during the early pandemic.

## Introduction

According to the WHO, suicide is the second leading cause of death among 15–29-year olds globally [1]. In Germany, suicides also still make up the largest group of non-natural deaths by far. Alone in the city of Frankfurt am Main (approximately 765,000 residents), there have been around 90 suicides per year in recent years. Although the number of suicides in Germany has dropped significantly since 1980, according to statistics from the German Federal Statistical Office, the number has stagnated at around 10,000 suicides per year since 2007 [2].

At the beginning of the COVID-19 pandemic, there were concerns that suicide rates might rise in response to the psychological burden of the pandemic, for example, through personal health fears, social isolation, unemployment, or a national economic downturn [3]. In the UK, in May 2020, in a press release, the Royal College of Psychiatrists even forecast a “tsunami of mental illness” in response to pandemic-related stresses [4]. In an overview of 68 publications, of which 65% had looked at the period of the first wave of COVID-19 infections and the summer plateau in 2020, Mauz et al. did not, however, find this expectation corroborated for Germany; instead, the evidence at the time did not allow conclusions about the frequency with which people developed mental disorders during the pandemic [5]. A British study by Marzano et al., moreover, found that reports of a rise in suicides during the early months of the pandemic were by and large inaccurate and reflected poor reporting standards in some of the media [6]. There have also been some authors who have even contended that increased feelings of social cohesion and resilience during the pandemic might actually serve to lower the risk of suicide [7, 8].

The suicide prevention program “FraPPE” (FKZ: ZMVI1-2517FSB136, https://frappe-frankfurt.de/frappe) is a communal multi-center, multi-level intervention study in which, on the one hand, the previous activities of a pre-existing regional suicide prevention network (Frankfurt Network for Suicide Prevention, FRANS) (e.g., destigmatization campaigns) were expanded and tested for their effectiveness. On the other hand, further evidence-based measures were established and evaluated. These are, for example, specific interventions in psychiatric hospitals, gatekeeper trainings, or strengthening the help networks. Among others, the participating institutions were different Departments of the University Hospital as well as the communal health authority. The primary objective was to reduce the number of suicides by 10% per year during the span of the project (November 2017–December 2020). A secondary project objective was to lower the number of suicide attempts.

Because the early period of the COVID-19 pandemic still coincided with the FraPPE program’s runtime, the medicolegal data collected in the context of this program could be used to assess whether the beginning of the pandemic and the concomitant lockdown measures led to a rise in suicides compared to a similar observation period from the previous, pre-pandemic, year (baseline). The goal of the present sub-study in the context of the FraPPE project was to compare the prevalence and characteristics of all completed suicides in the city of Frankfurt am Main for a 10-month period before the pandemic (March 2019–December 2019) with one from the beginning of the pandemic (March 2020–December 2020). Partial results of the FraPPE project in regard to suicide attempts have already been published in a different sub-study [9].

## Material and methods

This sub-study is based on the evaluation of partial data gathered in the context of the FraPPE project. The inclusion criteria were all completed suicides in the city of Frankfurt am Main for two observation periods, May 2019 to December 2019 (baseline) and March 2020 to December 2020. The data were collected in collaboration with the municipal health department and the criminal investigation department. The data stemmed from site inspections, questioning of relatives, criminal investigation results, autopsy results, and chemical and toxicological analysis results. Suicides were classified as completed suicides based on the entirety of findings, irrespective of whether the individual had died at the suicide site or had died later on (even with considerable delay) as a result of the suicide attempt. The autopsies had either been court-authorized or had been done with the consent of the next-of-kin, or others with the right of disposition, after the body had been released by the prosecution authority. Chemical and toxicological analyses were performed on evidentiary tissue samples taken at autopsy.

To allow comparison, the data for the two observation periods, May 2019 to December 2019 (baseline) and March 2020 to December 2020, were transferred to Microsoft^©^ Excel^®^ and then evaluated with descriptive statistics using R version 3.6.1 (R Core Team 2019) and R Studio version 1.2.5 (R Studio Team 2020). Mean values and median values were determined for numerical variables; binary and categorical variables were described in terms of frequencies and percentages. A significance level of alpha = 0.05 (5%) was assumed for the statistical tests, and two-sided hypothesis testing was used. Non-parametric tests were used for variables without normal distribution. The exact test of the Chi-square (*χ*^2^) statistic was used when expected cell counts were less than 5. For *p*-value calculation of the incidence rate ratio (IRR), we used the exact Poisson test.

## Results

### Absolute changes in suicide numbers and rates

**Fig. 1:**
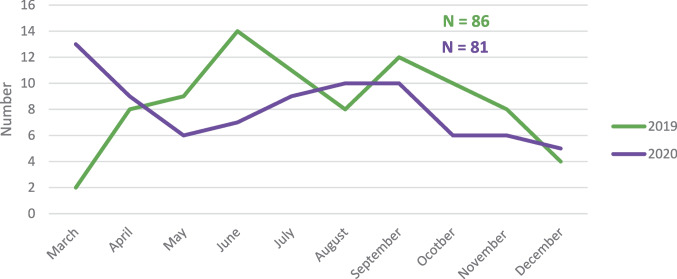
Number of suicides in both observation periods (before and during the early months of the pandemic)

**Fig. 2:**
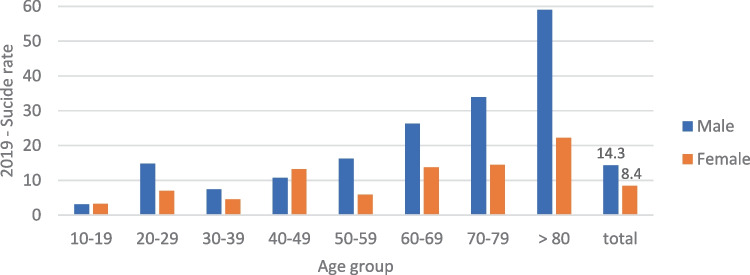
Suicide rate per 100,000 inhabitants, according to age and sex, “total” includes all age groups, before the pandemic (March–December 2019)

**Fig. 3:**
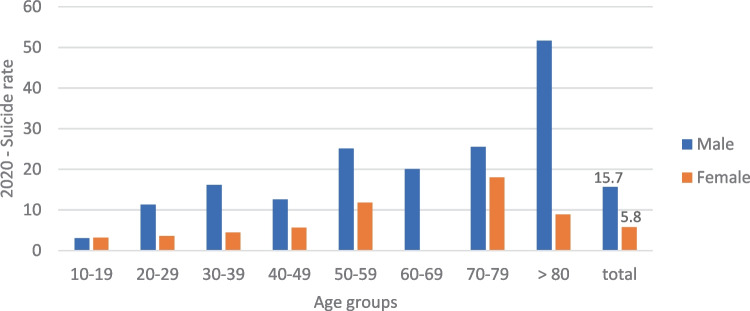
Suicide rate per 100,000 inhabitants, according to age and sex, “total” includes all age groups, at the beginning of the pandemic 2020 (March–December 2020)

For the baseline observation period between March and December 2019, 86 completed suicides had been registered in Frankfurt am Main. In comparison, for the observation period during the early months of the pandemic, between March and December 2020, there had been 81 completed suicides. This finding did not, however, constitute a statistically significant difference in absolute suicide numbers or in suicide rates (see Figs. 2 and 3) between the two periods (incidence rate ratio (IRR) = 0.94, *p* > 0.05). It must, however, be kept in mind that this result may be influenced by the comparatively small case numbers. Notwithstanding, there was a notable difference in suicide numbers between the two observation periods for the month of March (2 cases in 2019; 13 cases in 2020). Figure 1 depicts the distribution of cases for each month during the two observation periods.

### Age and sex distribution

Table 1 provides an overview of the age and sex distribution of the suicides in both of the observation periods. The observed proportional shift towards male sex at the beginning of the pandemic did not, however, prove to be statistically significant (Chi^2^(1) = 1.93, *p* > 0.05, OR = 1.69). Although the analysis of suicide rates in the respective age groups did show an increase in suicide rate per 100,000 residents with increasing age, there was no statistically significant difference between the two periods (Poisson regression with number of suicides as the dependent variable, study period as the interaction term, and age group as the explanatory variable) (Figs. 2 and 3).

**Table 1:** Age and sex distribution in both observation periods (before and during the beginning of the pandemic)

	March 2019– December 2019		March 2020–December 2020	
	Men	Women	Men	Women
Number of suicides	54	32	59	22
Proportion (%)	62.8	37.2	72.8	27.2
Mean value age-at-death (years)	55.0	54.3	52.6	54.3
Median age-at-death (years)	58.0	52.5	54.0	51.5
Youngest suicide (years)	16	16	16	17
Oldest suicide (years)	96	90	87	89

### Nationality

**Fig. 4:**
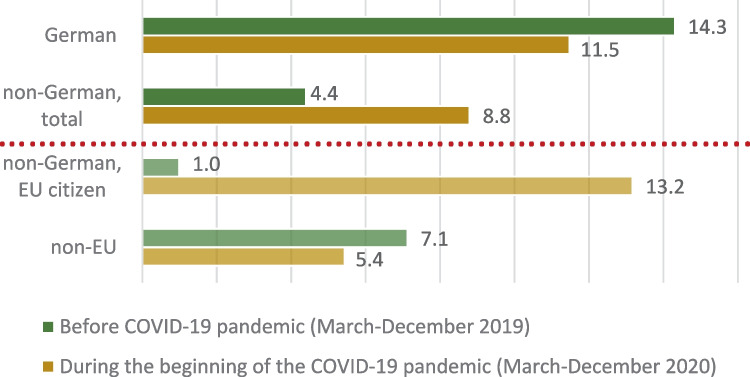
Suicide rate per 100,000 inhabitants according to nationality/country of origin

Figure 4 illustrates the relationship between suicide rate and nationality. Although a nationality could be established for all suicides, dual citizenships were not recorded in our case collective. The suicide rate for non-German nationals rose from 4.4/100,000 inhabitants to 8.8/100,000 inhabitants (IRR = 1.99, *p* > 0.05) at the start of the pandemic. Notably, this rise in suicide rate was statistically significant within the group of non-German EU citizens (IRR = 13.3, *p* < 0.001). In contrast, within the group of German citizens, the suicide rate sank from 14.3/100,000 to 11.5/100,000 inhabitants (IRR = 0.8, *p* > 0.05); this difference was, however, not significant.

### Suicide method

**Fig. 5:**
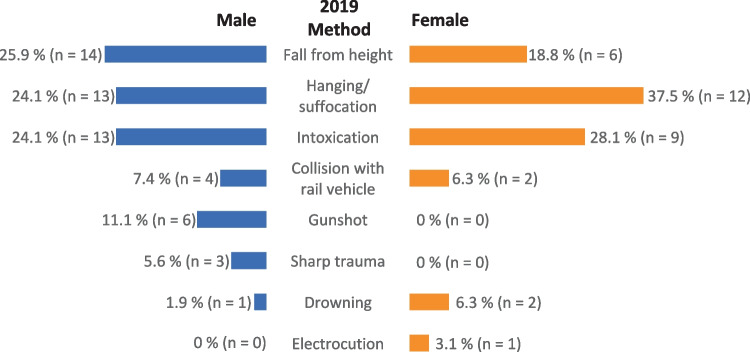
Suicide methods according to sex before the pandemic (March–December 2019)

**Fig. 6:**
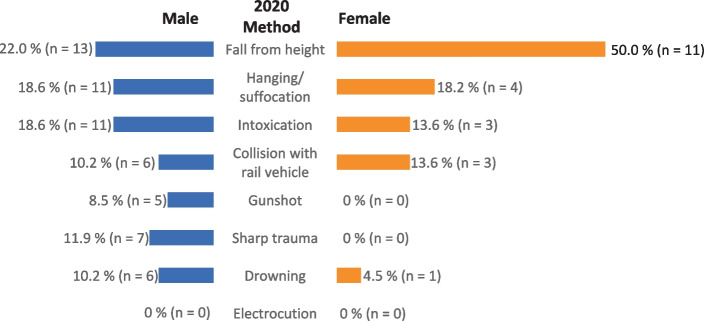
Suicide methods according to sex at the beginning of the pandemic (March–December 2020)

Figures 5 and 6 show the distribution of suicide methods in relation to sex. In regard to the three most commonly used suicide methods, no differences were found for men between the two examined periods: fall from a height (26% vs. 22%), followed by intoxication and hanging/suffocation (24% vs. 19% each). For women, however, there was a shift in the choice of methods from hanging/suffocation (38%), intoxication (28%), and fall from a height (19%) before the pandemic to fall from a height (50%), hanging/suffocation (18%), and intoxication and collision with a railway vehicle (14% each) in the early months of the pandemic. However, likely due to the small case numbers within the respective categories, these shifts did not prove to be statistically significant.

### Known mental disorders

Table 2 presents the distribution of the main diagnoses related to mental and behavioral disorders among the suicides. In both of the examined periods, 40% of the suicides had not been known to have a mental disorder. Before the pandemic, 54% of the suicides were known to have a mental illness in contrast to 44% during the early pandemic. In around 7%, respectively, 14% of the cases, this information could not be determined, e.g., no next-of-kin who could be questioned at the suicide scene or contacted later on. The majority of main diagnoses were from the category of mood (affective) disorders (F3x).

**Table 2:** Main ICD-10 diagnoses in the category of mental, behavioral, and neurodevelopmental disorders for suicides during the baseline and the pandemic observation period

Main diagnosis according to ICD-10 category (%)	March 2019–December 2019	March 2020–December 2020
No known diagnosis	39.0	41.9
F0x (mental disorders due to known physiological conditions)	1.2	1.2
F1x (mental and behavioral disorders due to psychoactive substance use)	7.0	11.1
F2x (schizophrenia, schizotypal, delusional, and other non-mood psychotic disorders)	9.3	11.1
F3x (mood (affective) disorders)	33.7	27.2
F4x (anxiety, dissociative, stress-related, somatoform and other nonpsychotic mental disorders)	5.8	1.2
F5x (behavioral syndromes associated with physiological disturbances and physical factors)	5.8	1.2
F6x (disorders of adult personality and behavior)	7.0	4.9
F7x (intellectual disabilities)	0.0	1.2
Unknown	6.9	13.5

## Discussion

The effects of the COVID-19 pandemic on mental health are the subject of current research. In this context, the present study set out illuminate possible effects of the COVID-19 pandemic on suicide rates and patterns in Frankfurt am Main, Germany, by analyzing data that had been gathered within the scope of the FraPPE suicide prevention project.

In terms of absolute numbers, no significant difference in the number of suicides was found between the two observation periods. A comparison of the suicide numbers for Germany as a whole with the numbers found in our study also did not show a significant increase in suicides during the investigated periods (7561 suicides in 2019 versus 7655 in 2020). Similarly, a study by Wollschläger et al. [1], in which the researchers compared suicide rates in the federal state of Rheinland-Pfalz with those from a comparable region in Italy for 2011–2019 and 2020, also did not find statistically significant differences in suicide rates for either of the two regions. Furthermore, in a study that evaluated data collected between 2017 and 2021 for the German federal states of Rheinland-Pfalz, Sachsen, and Schleswig–Holstein, Radeloff et al. also did not find statistically significant evidence for a rise in suicide numbers during the COVID-19 pandemic [10]. Although Radeloff et al. did observe absolute and relative rises and declines in suicide frequencies within specific age groups, when the data were stratified for age and sex, no larger pattern emerged [10].

These observations are all in accord with the results of a recent, large-scale meta-analysis by Pirkis et al. [11], which investigated data from 33 countries, to assess whether the number of suicides had risen during the COVID-19 pandemic. This meta-analysis also contained data from the FraPPE project. Pirkis et al. did not find a significant increase in suicide rates in the majority of the evaluated countries. On the contrary, in keeping with findings from other studies, the evidence suggested that the suicide rates had dropped in many countries [10, 12, 13]. The few countries in which Pirkis et al.’s meta-analysis pointed to a potential rise in suicide numbers were Japan, Austria, and the Czech Republic [11]. Although a study by Sardar et al. also showed an increase in suicide numbers in India between April and June 2020 [14], the study collective in Sardar et al.’s study and the one in our sub-study can only be compared to a limited extent due to the large socio-economic differences between India and Germany, respectively, India and Frankfurt am Main. In connection with that point, the study by Pirkis et al. stated that despite the lack of data on suicide numbers in lower-middle-income countries, their analyses suggested that such countries fared less well [11]. This can lead to the assumption that, conversely, Germany as a high-income country benefits from good social and economic systems which could have had a greater impact on the suicide rates than it had been in low- or middle-income countries. Nevertheless, it should be taken into consideration that these effects may have become weaker during the pandemic and therefore increases in suicide numbers may be delayed [15]. Another aspect which may determine variations in suicide rates in general may be the impact of government decisions on ethical issues. Law decrees issued by governments, imposing measures limiting social contacts, stopping non-essential production activities, and restructuring public health care, e.g., in order to privilege assistance to COVID-19 patients, may have tended to affect even countries with limited public health care resources [16].

The main goal of the FraPPE project was to reduce the number of suicides by 10% per year during the project’s runtime by implementing a bundle of measures. This bundle comprised suicide prevention and postvention measures both on an individual level, for individuals who had attempted suicide, and on a health care professional and city gatekeeper level. The project program further included anti-stigma and awareness measures. Evidence-based evaluation of the implemented measures was also conducted to assess their efficacy. In addition, the program monitored suicide methods to be able to respond quickly where possible, for example, with construction-related safeguards. McCartan et al. suggest that such preventive measures may be suitable as deterrents to discourage people with a potentially heightened risk for suicide from attempting suicide [13]. In our present study, both the briefness of the period in which the preventive FraPPE measures were implemented, and the small number of cases must be viewed as critical limitations. Therefore, the absence of a rise in suicides in Frankfurt am Main during the first months of the pandemic cannot conclusively be attributed to the success of the FraPPE preventive measures. The possibility remains that, irrespective of these measures, the consequences of the COVID-19 pandemic in terms of a rise in suicide rates may still only become apparent with considerable delay [17]. In this sense, it would be expedient to continuously keep monitoring suicide rates around the world to allow swift action if rises in suicides become apparent [18].

In regard to sex distribution among our suicide cases, there appeared to be a shift towards male suicides during the COVID period, but this shift did not prove to be statistically significant. There were also no significant differences between the two study periods in terms of age distribution. This finding is in accord with those from other German studies [1, 10, 19].

In terms of the relationship between nationality and suicide numbers, there was a statistically significant shift between the two periods towards an increase in suicides among non-German EU citizens during the pandemic. A limitation of our study is that despite the interdisciplinary cooperation, the respective socio-economic status could often not be clarified. Nevertheless, various studies indicate differences between people with a migration background compared to people without a migration background. On average, people with a migration background have a lower net income than people without a migration background [20]; Rommel et al. showed that depressive symptoms are more frequent in men as well as in women with a migration background than in people without a migration background [21]. And Teltemann et al. examined the reasons for the spatial segregation of families with a migration background in large German cities and showed that both socio-economic status and discrimination processes do have an influence [22]. Similar demographic findings to the findings of the present study are reported in a study from the USA [23], in which the age-adapted suicide rate among non-white persons was found to be 62% higher (under the constraints of small case numbers) at the beginning of 2020 than for the previous year. Even if multifactorial reasons must be assumed here, the results might nonetheless suggest an increased vulnerability and uneven outcome among ethnic minorities, amid factors such as an increased risk for infection with SARS-CoV-2, increased mortality rates—also of family members—and economic disadvantages [24, 25]. A further explanation for this finding may also be the higher structural barriers to accessing psychosocial facilities for these groups of people.

Although no statistically significant differences were found between the two observation periods in our study in terms of suicide methods used by women due to the low case numbers, a notable finding was, nonetheless, that the preferred suicides methods used by women during both periods were hard methods, rather than the softer suicide methods frequently described for women in the literature [12, 26–28]. The circumstance that the female collective from Frankfurt am Main often chose fall/jump from a height as a suicide method may be explained by the availability of tall buildings in this city. This finding also points to the possibility of securing such buildings with suicide-preventive constructions. A further possible explanation for the rise of this suicide method among women during the pandemic could also be that the contact restrictions and lockdown measures led to more time spent at home. Further geographical analysis of the study collective is ongoing, in particular, in respect to identifying possible preventive approaches.

No differences in the distribution pattern of pre-existing mental disorders or diagnoses were found between the two periods. A notable observation, nonetheless, was that a large proportion of the suicides had not been known to have mental disorders or diagnoses. This finding is contrary to the findings of other studies so far, which even report a prevalence of up to 90% for psychiatric disorders within their suicide collectives [29, 30]. In the context, it is of little surprise that most of these mental disorders were found to be in the category of affective disorders [30]. This was similarly the case in our suicide collective.

All in all, although our study did not find a statistically significant rise in suicides in Frankfurt am Main during the early months of the COVID-19 pandemic, a trend towards more male and more non-German suicides, as well as a shift in the suicide methods chosen by women, was observed.

## Conclusion

The present evaluation of a part of the data collected within the scope of the FraPPE suicide prevention project did not reveal an increase in suicide numbers or rates during the examined period in the early months of the COVID-19 pandemic. Nonetheless, the results still speak to the need for focused approaches to prevent suicide, in particular among disadvantaged groups in the population. Because pandemics or other crises (e.g., energy crisis) can always occur and are known to negatively affect people’s mental health, long-term, population-based monitoring of suicides is a useful approach to allow speedy interventions tailored to the needs of the target groups.

## Key points

1. The prevalence and characteristics of all completed suicides in the city of Frankfurt am Main were compared for a 10-month period before the COVID-19 pandemic with one during the early pandemic.
2. No statistically significant differences were found in terms of suicide numbers, suicide methods, age distribution, pre-existing mental disorders, or diagnoses.
3. With regard to sex distribution, there appeared to be a shift towards male suicides during the beginning of the COVID period.
4. There was a statistically significant shift between the two periods towards an increase in suicides among non-German EU citizens during the early pandemic.
5. The preferred suicide methods used by women during both periods were so called hard methods.
6. Long-term, population-based monitoring of suicides should be undertaken to enable rapid interventions tailored to the needs of target groups.

## Data Availability

The generated data sets that were analyzed in this study are available from the corresponding author upon reasonable request.
